# The synergistic effects of polyphenols and intestinal microbiota on osteoporosis

**DOI:** 10.3389/fimmu.2023.1285621

**Published:** 2023-10-23

**Authors:** Keyu Wang, Siwang Hu

**Affiliations:** ^1^ The Orthopaedic Center, The Affiliated Wenling Hospital of Wenzhou Medical University (The First People’s Hospital of Wenling), Wenling, Zhejiang, China; ^2^ College of Bioscience and Biotechnology, Hunan Agricultural University, Changsha, Hunan, China

**Keywords:** osteoporosis, gut microbiota, polyphenol, immune system, pathway

## Abstract

Osteoporosis is a common metabolic disease in middle-aged and elderly people. It is characterized by a reduction in bone mass, compromised bone microstructure, heightened bone fragility, and an increased susceptibility to fractures. The dynamic imbalance between osteoblast and osteoclast populations is a decisive factor in the occurrence of osteoporosis. With the increase in the elderly population in society, the incidence of osteoporosis, disability, and mortality have gradually increased. Polyphenols are a fascinating class of compounds that are found in both food and medicine and exhibit a variety of biological activities with significant health benefits. As a component of food, polyphenols not only provide color, flavor, and aroma but also act as potent antioxidants, protecting our cells from oxidative stress and reducing the risk of chronic disease. Moreover, these natural compounds exhibit anti-inflammatory properties, which aid in immune response regulation and potentially alleviate symptoms of diverse ailments. The gut microbiota can degrade polyphenols into more absorbable metabolites, thereby increasing their bioavailability. Polyphenols can also shape the gut microbiota and increase its abundance. Therefore, studying the synergistic effect between gut microbiota and polyphenols may help in the treatment and prevention of osteoporosis. By delving into how gut microbiota can enhance the bioavailability of polyphenols and how polyphenols can shape the gut microbiota and increase its abundance, this review offers valuable information and references for the treatment and prevention of osteoporosis.

## 1 Introduction

With the continuous progress of population aging, osteoporosis (OP) has become one of the top three chronic diseases (1). Osteoporosis is a chronic disorder characterized by the deterioration of bone tissue microstructure and loss of bone mass, primarily attributed to the up-regulation of osteoclasts (2). Osteoblasts are essential cells for bone growth and maintenance as they form bone tissue (3). Studies have shown that hormonal imbalance and local oxidative inflammation *in vivo* can affect the dynamic balance of osteoclasts and osteogenesis (4), mainly manifested as degradation of bone microstructure, reduction in bone mass, and decrease in bone strength (5). The decline in osteogenic differentiation and intraosseous angiogenesis of bone marrow mesenchymal stem cells occurs simultaneously, leading to increased bone fragility and susceptibility to fractures (6, 7).

Phenols or polyphenols found in our diets are incredibly abundant and can be found across a wide range of plants in nature. At present, more than 8,000 phenolic structures are known, of which more than 4,000 kinds of flavonoids have been identified (8). Polyphenols are chemically identified as compounds possessing phenolic structural characteristics. However, this diverse class of natural products encompasses various subgroups of phenolic compounds. Rich sources of polyphenols include fruits, vegetables, and whole grains, as well as other types of foods and drinks like tea, chocolate, and wine.

In addition, most plant polyphenols are in the form of glycoside, the skeleton of polyphenols has different positions with different sugar units and acylation of sugar. Hence, polyphenolic aglycones can be categorized into phenolic acids and flavonoids, and polyphenolic amides, based on their chemical structure (9). For example, quercetin is a well-known flavonol flavonoid that can be found in a variety of food sources, and its main form is glycoside (10).

Turmeric has been used throughout history as a spice, herb, and dye, and is widely used worldwide as an ingredient in curry powder. In recent decades, numerous studies have demonstrated the extensive array of advantageous characteristics associated with curcumin. These include anti-inflammatory, antioxidant, hypoglycemic, wound-healing, antibacterial, and antitumor activities (11). Curcumin is an important bioactive substance, which mainly exists in the rhizome of turmeric (12).

The phenolic hydroxyl structure of plant polyphenols has antioxidant activity, including direct and indirect antioxidant effects (13). In addition, polyphenols also inhibit osteoporosis through mechanisms such as anti-inflammatory and promoting bone formation (14). Most natural polyphenols must be absorbed and utilized under the action of specific gut microbiota, and phenolic metabolites may have activities that are not present in the original compounds.

Research has revealed that polyphenols interact with gut microbiota, thereby enhancing the functionality of the intestinal mucosal mechanical barrier (15). Polyphenols are capable of changing the composition of gut microbiota, which can improve the function of the intestinal mucosal mechanical barrier. Studies have revealed that Resveratrol, a natural polyphenol found in plants, may affect the intestinal barrier by inhibiting the growth of harmful bacteria and fungi, regulating the expression of tight junction proteins, and balancing pro-inflammatory and anti-inflammatory T cells. These mechanisms help to control the growth of pathogens and maintain the integrity of cellular barriers (16, 17). These actions help to prevent damage to the intestinal barrier and maintain its proper functioning. The activation of the PI3K/Akt-mediated Nrf2 signaling pathway by Resveratrol protects IPEC-J2 cells from oxidative stress, preventing damage to the intestinal barrier (18, 19).

On the other hand, recent studies have revealed that tea polyphenols can prevent the disturbance of gut microbiota by regulating gut microbiota (20, 21). Evidence suggests that epigallocatechin-3-gallate, the principal active component in green tea, exhibits the potential to alleviate inflammatory bowel disease by primarily targeting bacteria responsible for producing short-chain fatty acids, including Akkermansia (22, 23). Subsequently, these bacteria produce functional SCFAs which contribute to beneficial changes in the gut microbiome. These changes lead to increased production of protective SCFAs, such as butyrate, which trigger significant antioxidant, anti-inflammatory, and barrier-strengthening responses, ultimately reducing inflammation and damage in the gut (23, 24). Additionally, polyphenols play a “prebiotic” role in the gut, supporting the growth of beneficial bacteria. While the impacts of various plant polyphenols on the gut microbiota may vary, the majority of them typically stimulate the proliferation of beneficial bacteria (25, 26). It is worth noting that the health benefits of most plant polyphenols are achieved through a “two-way interaction” with intestinal microorganisms.

The gut microbiota is the body’s “second largest gene pool” and is the symbiotic, symbiotic, and disease-causing microbes that live in our gut (27). The gut microbiome contains approximately 1,200 bacterial species, with the main representative groups being Bacteroidetes, Firmicutes, Actinobacteria, Proteobacteria, and Myxococcus (28). The metabolites of gut microbiota can act on the human gut, thereby regulating and preventing most diseases. Among these factors, intestinal microbes crucially influence the balance of bone health by exerting effects on host metabolism, immune function, hormone secretion, and the gut-brain axis (29, 30). These interactions can contribute to the development of osteoporosis.

The intestinal barrier function is significantly influenced by the interplay between gut microbiota and the immune system. GM forms various symbiotic relationships with the host, including parasitic, commensal, and mutualistic relationships. Under normal physiological conditions, the gut microbiota contributes to food digestion, combats pathogens, and aids in the development of the host immune system, particularly during the early post-natal period. Throughout one’s lifespan, the gut microbiota interacts with the host, playing a role in modulating both gut and systemic immunity (31, 32).

The intricate interplay between immune cells and bone cells is closely intertwined, with the gut microbiota playing a vital role in maintaining bone health through its influence on bone turnover and density (33). By producing metabolites, intestinal microorganisms influence and regulate intestinal barrier function. A normal intestinal barrier is important for isolating harmful substances, facilitating nutrient absorption, and providing immune protection. Impaired intestinal barrier function is considered one of the pathogenic factors contributing to osteoporosis. The intestinal mucosal barrier is made up of four components: a mechanical barrier, chemical barrier, immune barrier, and biological barrier. Together, these barriers prevent harmful substances such as toxins and bacteria from entering the body through the intestinal mucosa (34). When the intestinal mucosal barrier is compromised, it can cause an increase in intestinal permeability. This can lead to bacterial and endotoxin translocation, which can trigger or worsen systemic inflammation and multiple organ dysfunction. Intestinal epithelial cells are closely arranged by cell junctions, which are composed of tight junctions, adhesion junctions, and desmosomes, which can effectively block the entry of bacteria, viruses, and endotoxins, and it is essential for nutrition absorption and immune function (35). The chemical barrier consists of gastric acid, bile, a wide range of digestive enzymes, lysozyme, mucin, and bacteriostatic substances produced by commensal bacteria residing in the intestinal cavity. It has the effect of inactivating pathogenic microorganisms (36). The immune barrier is composed of intestinal mucosal lymphoid tissue, cells, and secreted antibodies on the surface of the intestinal mucosa, which induce local and systemic immune responses and protect the intestinal tract from damage by foreign antigens and abnormal immune responses (37). The biological barrier is mainly composed of normal gut microbiota, which is the intestinal normal parasitic flora with colonization resistance to foreign strains. When the stability of this microflora is disrupted, the intestinal colonization resistance is significantly diminished, thereby increasing the risk of potential pathogens, including opportunistic pathogens, colonizing and invading the gut (38). Dysfunction of the gut microbiota can lead to impaired intestinal barrier function, causing the absorption of harmful substances and inflammation, ultimately resulting in bone loss, inhibited osteoblast growth, and increased osteoclast activity (39, 40)

**Figure 1:**
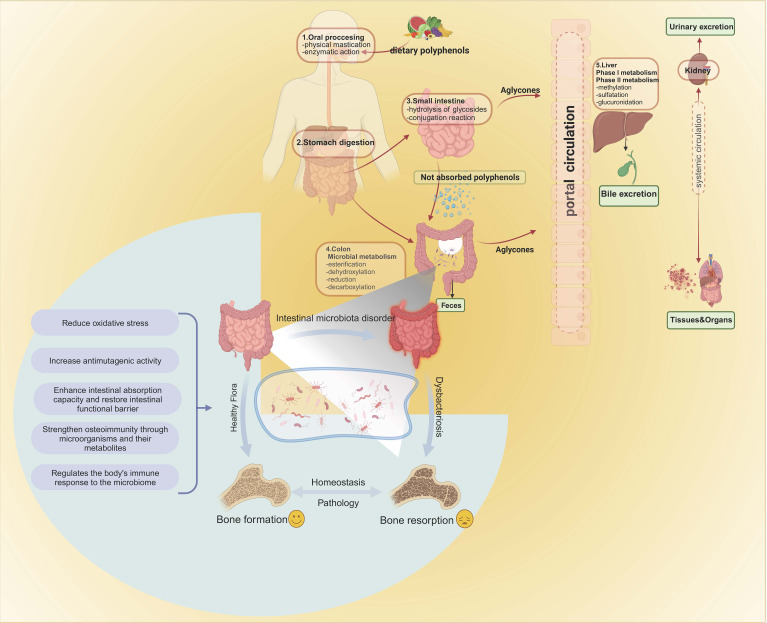
Absorption and metabolism of foodborne polyphenols. Intestinal enzymes and gut microbiota are involved in the metabolism and absorption of polyphenols in the intestine. Once converted, the polyphenols travel to the liver through the portal vein, where they undergo two metabolic stages, resulting in different metabolic compounds. These compounds then enter phase II metabolism in the circulatory system, where sulfate, glucuronide, and methyl conjugates are produced. These conjugates can be detected in urine several days after ingestion. Gut microbiota act on bone, leading to bone resorption or inhibiting osteoporosis mechanisms. Bone formation and bone resorption are key factors affecting the pathogenesis of osteoporosis. Intestinal microbes can affect bone growth by regulating intestinal homeostasis, such as reducing oxidative stress, increasing anti-mutagenesis activity, enhancing intestinal barrier function, and regulating immune response. Intestinal microbes always maintain the homeostasis of the intestinal environment and play a role in the prevention and treatment of osteoporosis.

Chronic inflammatory diseases and immune dysfunctions have been associated with a higher incidence of osteoporosis, primarily attributed to the excessive production of pro-inflammatory cytokines that stimulate osteoclastic activity. Consequently, GM disorders weaken intestinal barrier function, and enhanced immune system reactivity contributes to the entry of harmful substances into the body (39), thereby promoting the production of factors that activate osteoclasts and lead to bone resorption, ultimately causing osteoporosis. Therefore, gut microbiota can affect bone formation and bone resorption **Figure 1**.

Through the interaction of polyphenols with gut microbiota, intestinal barrier function can be enhanced, and simultaneously, the richness and activity of the gut microbiota increase (41, 42). Gut microbiota converts polyphenols from food into more bioavailable microbial metabolites. Therefore, under the synergistic effect of the two, the effect of each on the treatment of osteoporosis is maximized (43).

## 2 Interactions between polyphenols and gut microbiota

### 2.1 Effects of GM on foodborne polyphenols

Polyphenols are renowned for their antioxidant properties and are frequently utilized in the treatment of diverse diseases. Their metabolic degradation in the body is influenced by GM (43).

**Figure 2:**
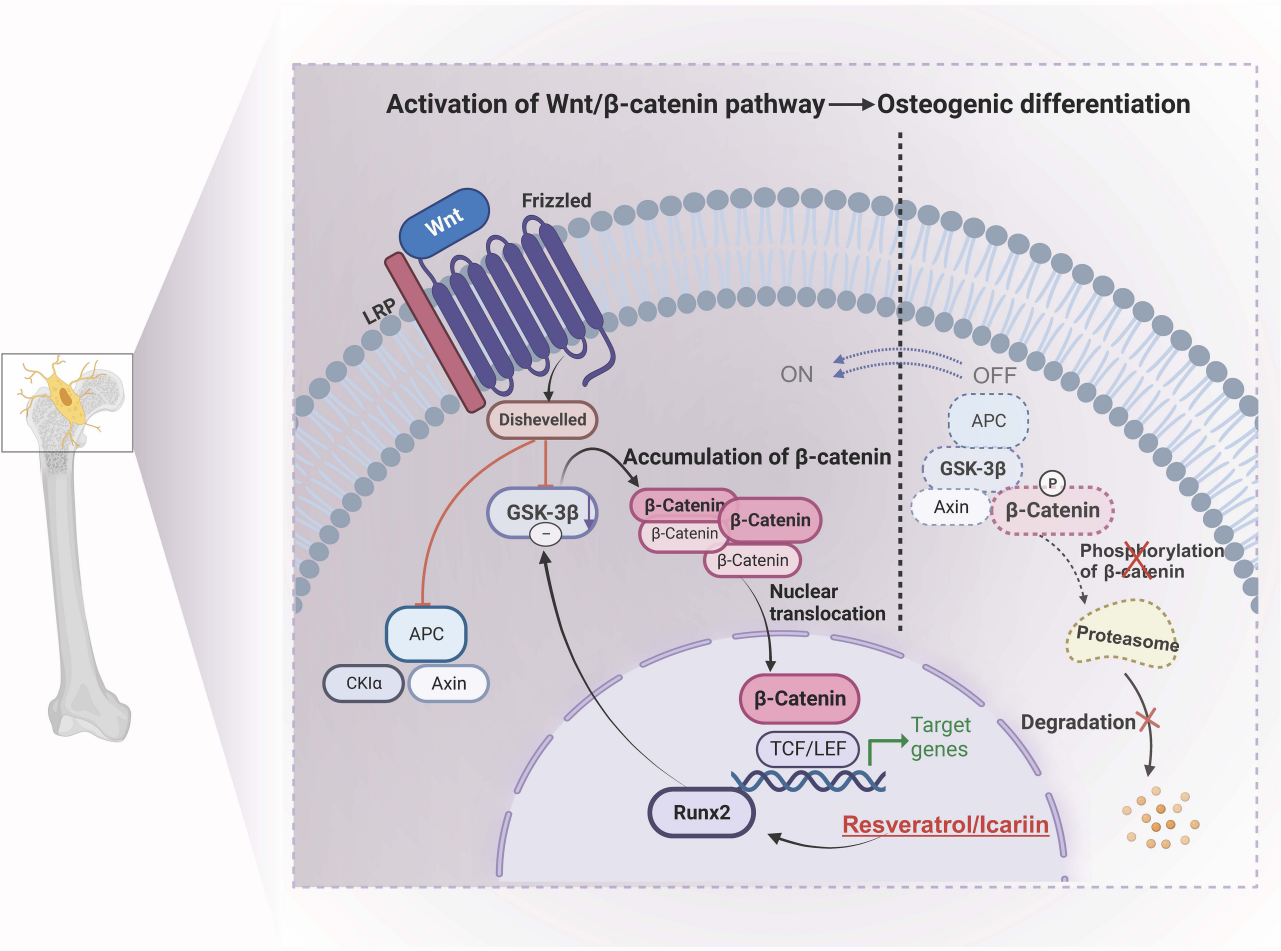
Molecular mechanism of osteogenic differentiation controlled by resveratrol and icariin. Icariin down-regulates the expression level of GSK38 by stimulating the expression of Runx2 through veratrole, thus achieving stable accumulation of β-catenin and transferring into the nucleus, thereby activating Wnt/B-catenin pathway, promoting osteogenesis differentiation and enhancing bone density. GSK-3β, Glycogen synthase kinase 3β; APC, adenomatous polyposis coli; Runx2/TCF/LEF, specific transcription factors.

Polyphenols, when consumed through food, are present in the form of glycosides and complex oligomerization structures. In the human body, these complex structures undergo sequential metabolism. After ingestion, some polyphenols are minimally absorbed in the stomach, primarily as phenolic acids (44). Only a small fraction (5-10%) of polyphenols are absorbed in the small intestine, primarily in the form of free polyphenols (45). Under the influence of intestinal microbial flora, the polyphenols that remain unabsorbed, especially the ones that are bound, are transported to the colon where they undergo decomposition, release, and subsequent absorption. **Figure 2** shows the absorption and metabolism of foodborne polyphenols.

Polyphenols exhibit a range of structural variations that influence their bioavailability. Upon ingestion, these compounds tend to accumulate in the large intestine where they undergo extensive metabolism by gut microbiota. The microbiota transforms polyphenols into metabolites, making them bioactive. The extended retention of polyphenols in the intestines can yield beneficial effects on the gut microbiota. On the contrary, gut microbiota plays a crucial role in enhancing the biological activity of polyphenols by converting them into active metabolites known as phenolics (46). Polyphenols and other compounds are biotransformed by various bacterial species, including Bifidobacterium, Lactobacillus, Escherichia coli, Bacteroides, and Eubacterium, resulting in the production of short-chain fatty acids (SCFAs) and other metabolites (47). Short-chain fatty acids (SCFAs) play a significant role in reducing the pH of the intestines, suppressing the growth of harmful pathogens, and facilitating optimal absorption of minerals and vitamins. The metabolism of polyphenols in the intestines is carried out by microorganisms, which utilize hydrolysis, lysis, and reduction mechanisms to break down the polyphenols (48). Phenolic compounds in food and herbal products exist as conjugates and require hydrolysis for absorption (49). Phenolic compounds like hesperetin, daidzein, ellagic acid, caffeic acid, and secoisolariciresinol must undergo hydrolysis to produce phenolic aglycons that can be absorbed (50). Metabolites can undergo two processes, either being metabolized in the gut or absorbed directly. During the cleavage process, the carbon ring is opened and the C-C bond is broken, while methyl ether is removed through demethylation. Hydrolase then releases glycogen, which is subsequently broken down through the cleavage of flavonoids’ carbon ring, the removal of ellagic acid’s esterification through lactone ring opening and decarboxylation, and the cleavage of the quinic acid ring from chlorogenic acid (50). C-ring cleavage converts isoflavone daidzein to O-demethylancomycin and flavonoid hesperidin to 3-(30-hydroxy-40-methoxyphenyl) hydroxy acrylic acid. Collectively, these substances facilitate the transformation of non-absorbable oligo procyanidins into easily assimilable phenolic acid molecules, such as derivatives of hydroxyphenylacetic acid, hydroxyphenylpropionic acid, and hydroxyphenylvaleric acid. During the reduction process, gut microbiota also catalyze different PPs reduction reactions (51). The transformation of caffeic acid into 3,4-dihydroxyphenylpropionic acid is a typical hydrogenation reaction (52). The implementation of targeted dehydroxylation processes can lead to the production of monohydroxy derivatives. This process also facilitates the conversion of the aliphatic side chain, resulting in the formation of phenylacetic acid, benzoic acid, and decarboxylated metabolites.

Studies have also shown that gut bacteria can metabolize resveratrol precursors, such as piceid, into resveratrol, thereby increasing its bioavailability. Bifidobacterium and *Lactobacillus* acidophilus are two specific bacteria responsible for producing resveratrol from piceid (53). Resveratrol, a polyphenol, has the ability to undergo glycosylation in the gut, resulting in its transformation into piceid. Piceid can then be absorbed in both its free and conjugated forms, the latter being referred to as piceid glucuronide. It is evident that polyphenol metabolites, which are metabolized by gut microbiota, exhibit a higher level of activity and are more efficiently absorbed.

### 2.2 Effects of polyphenols and their metabolites on GM

Polyphenols can impact the gut microbiota in two ways, by promoting the proliferation of beneficial bacteria and increasing their abundance. Polyphenols have the ability to mimic prebiotics and change the composition of the human gut microbiota. This has been shown through numerous studies, both *in vitro* investigations utilizing human gut microbiota and *in vivo* clinical trials. Foods rich in polyphenols have been consistently proven to effectively modify the gut microbiota. They achieve this by promoting the proliferation of beneficial bacteria such as Lactobacillus and Bifidobacterium. For example, cocoa polyphenols have been shown to regulate the composition of the gut microbiota, functioning through a probiotic mechanism (54). Cocoa polyphenols have the potential to stimulate the growth and proliferation of beneficial gut bacteria, including *Lactobacillus* and *Bifidobacterium*, while concurrently diminishing the population of harmful bacteria, including *Clostridium perfringens*.

Some tannins catabolites of the gut microbiota may have “prebiotic” activity, such as urolithin produced by pomegranate ellagitannins, which in preclinical studies has actively regulated lactic bacteria, *Bifidobacteria* and enterobacteria model of intestinal inflammation in rats (55).

In the human study, it was observed that a particular subset of the population (16 out of 20 subjects) exhibited a higher abundance of *Akkermansia muciniphila* in their gut microbiota both before and after the intervention. Notably, these individuals were capable of producing urolithin A (56). Another study conducted by the same research group further concluded that the consumption of pomegranate extract promotes the abundance of A. muciniphila (57).

In addition to the probiotic effects described above, Polyphenols have the ability to modulate the gut microbiota in a way that promotes the growth of beneficial strains, thereby positively impacting the overall health of the host, and the metabolites derived from polyphenols can enhance gut health and exhibit anti-inflammatory properties. For example, the bioactive metabolites of cocoa can enhance gut health, show anti-inflammatory effects, have a positive effect on the immune system, and reduce the risk of various diseases (58). Intake of polyphenols may improve the health effects of the gut microbiota by promoting the excretion of short-chain fatty acids, enhancing intestinal immune function, and other physiological processes.

a majority of studies have consistently demonstrated that polyphenols can induce favorable alterations in the gut microbiota composition. Specifically, when individuals consume a diet rich in polyphenols, notable changes occur in the human gut microbiota, the numbers of *Lactobacillus*, *Bifidobacterium*, *Akkermansia*, *Enterococcus*, and *Bacteroides* increase, while the ratio of enterococcus, Clostridium, and firmicutes to *Bacteroides* significantly decreases. As an example, red wine, which is rich in polyphenols, has been found to stimulate the growth of certain bacteria species, such as Bacteroides and Roseburia intestinalis, in the gut microbiota (59). Some types of polyphenols found in fermented papaya juice, such as gallic acid and caffeic acid, can affect the composition of the microorganisms in the intestines. Studies have shown that these polyphenols can decrease the number of harmful bacteria, like Enterococcus, Clostridium perfringens, and Clostridium difficile, while promoting the growth of beneficial bacteria, like Bifidobacterium. Moreover, certain polyphenols can also encourage the growth of fungi in the gut microbiota (60). Consuming grape seeds rich in procyanidins can increase the number of *lactobacilli*, *Clostridium*, and *Ruminococcus* in the gut (61). Vegetables, similar to fruits, are rich in polyphenols and prebiotic fiber. Dietary polyphenol intake in carrots can increase *Bacteroides* and *Lactobacillus*, and decrease the number of Bordetella such as *Clostridium perfringens*, *Clostridium coccoides*, *Bacteroides coccoides*, and *Enterobacterium ecium (* 62). In the study conducted by Xu Song et al., the impact of resveratrol on the intestinal biological barrier was investigated using 16S rRNA and metagenomic sequencing analyses. The findings revealed that resveratrol had a positive effect on the diversity and structure of the gut microbiota. Specifically, it increased the abundance of probiotic bacteria and regulated the function of the gut microbiota to counteract immunosuppression (63).

Monica Maurer Sost et al. conducted a study to evaluate the impact of citrus fruit extracts containing polyphenols hesperidin and naringin on the regulation of gut microbiota composition and activity using an *in vitro* model of colon dynamics. Their findings revealed that polyphenol hesperidin led to a dose-dependent increase in the abundance of *Roseburia, Eubacterium ramulus*, and *Bacteroides eggerthii* (64).

Tart cherry polyphenols underwent a bacterial fermentation assay *in vitro* and were subsequently assessed using 16S rRNA gene sequencing and metabolomics. *In vitro*, tart cherries were discovered to stimulate a significant rise of Bacteroides, possibly attributable to the presence of polysaccharides (65). In the human study, the consumption of tart cherries was linked to two distinct and contrasting responses, which were associated with the initial levels of Bacteroides (65). Studies have shown that individuals with a high initial abundance of *Bacteroides* in their gut microbiota tend to exhibit a specific response to tart cherry juice consumption. In this group, tart cherry juice consumption was associated with a decrease in Bacteroides populations and an increase in fermentative *Firmicutes*. Additionally, there was an observed increase in the presence of *Collinsella*, which has the potential to metabolize polyphenols. On the other hand, individuals with a low initial abundance of *Bacteroides* in their gut microbiota exhibited a different response to tart cherry juice consumption. In the group that consumed tart cherry juice, there was an observed increase in the populations of *Bacteroides* or *Prevotella*, as well as a rise in *Bifidobacterium*. Conversely, there was a decrease in the abundance of *Lachnospiraceae*, *Ruminococcus*, and *Collinsella* in these individuals, as indicated by the 16S rRNA gene sequencing and metabolomics analysis (65, 66).

Polyphenols, similarly to legumes, represent one of the primary bioactive compounds. *In vitro* research has demonstrated that germinated lentil seeds harbor potent antimicrobial compounds, including cysteine-rich peptides, which exhibit activity against detrimental microorganisms like E. coli and Staphylococcus aureus. In a study focused on mung bean coats, researchers performed simulated digestion and colonic fermentation *in vitro* to investigate the liberation of polyphenols from the mung bean coat and assess their bioactive properties. These experiments aimed to understand how the polyphenols in mung bean coats are digested and fermented within the gastrointestinal tract and their potential effects on human health. In the study involving the mung bean coat, during the process of colonic fermentation, a noteworthy enhancement in the relative abundance of beneficial bacteria, particularly Lactococcus and Bacteroides, was observed. This observation implies that the polyphenols released from the fermentation of mung bean coats potentially exert a favorable influence on the growth and multiplication of these beneficial bacterial populations within the colon (67). Studies exploring the effects of red wine polyphenols on intestinal microbiota have observed an increase in the concentration of specific bacterial genera in the intestines. Specifically, the *genera Clostridium*, *Bacteroides*, *Enterococcus*, and *Bifidobacterium* are positively influenced by red wine polyphenols. The findings indicate that the intake of red wine polyphenols could potentially yield advantageous effects on the composition of the intestinal microbiota (68), that is, accelerate the growth of “phoenixes”, *Klebsiella, Bacillus, Bordetella* and *Staphylococcus*, while reducing the growth of *Bacteroides*, *Clostridium*, anaerobic coccus, and Bifidobacterium. The incorporation of tea polyphenols, specifically catechins, in a culture medium containing human fecal bacteria was observed to lead to a reduction in the levels of harmful bacteria, including E. coli, Clostridium perfringens, and Bacteroides. This finding suggests that the introduction of tea polyphenols in the gut environment may have the potential to combat the proliferation of these specific pathogenic bacteria. The results of this study highlight the positive influence of tea polyphenols on the balance of gut microbiota. Ma et al. researched the impact of green tea polyphenols on the redox status of the intestine and its correlation with gut microbiota (69). It was found that *Spirochaetaceae* and *Bacteroides* were identified as biomarkers of intestinal redox status, revealing the benefits of tea polyphenols. The polyphenol compounds in oolong tea are mainly catechins, which can increase the number of Bacteroides, *Bifidobacterium*, and *Lactobacillus genera*. In a 2015 study, researchers examined the potential impact of saponins found in herbal teas on the gut microbiota of mice (25, 70). In the treatment group, the administration of ginseng, red ginseng, Panax San Qi, and ginsenosides led to noticeable increases in Enterococcus, Lactobacillus, and Bifidobacterium. In addition, a significant increase in the proportion of Firmicutes/Bacteroides was observed after consumption of Asarum and San Qi. Consumption of hypericum tea also increased the growth of aromatic coccus. The main phenolic substances in coffee are flavanols and chlorogenic acids (71). When male Wistar rats were fed coffee grounds, the number of microflora in *Ruminococcaceae, Muribaculaeceae, and Lachnospiraceae* increased, while the ratio of *Firmicutes* to *Bacteroidetes* decreased (72). Nuts are rich in polyphenols, mainly persimmonic acid and procyanidins (73). Increasing the intake of nut polyphenols can enhance the probiotic effect and benefit the gut microbiota. Ellagic tannins are metabolized into urolithin, which circulates in the plasma, thereby increasing the number of *Bifidobacterium* and *Lactobacillus* (74). From this, polyphenols play a probiotic role in the gut, shaping the gut microbiota and interacting with the gut microbiota. **Table 1** summarizes recent research findings on the effects of specific polyphenols and/or polyphenol-containing dietary sources on gut microbiome composition.

**Table 1:** Microbes provoked or inhibited in the gut based on the consumption of polyphenols.

Type of Polyphenol	Experimental method		Changes in the microbiota	Ref.	
Green tea polyphenols (catechins, flavonoids and flavonols)	*In vivo* experiment: Mice were divided into groups and given 100 mg/kg body weight TP (TPL), 200 mg/kg body weight TP (TPM), and 400 mg/kg body weight TP (TPH) by tube feeding, respectively, for 12 weeks		*C. Difficile↑* *C. Bacillus perfringens↑* *Bacteroides* spp.*↑*	(75)	
Polyphenols (saponins) in herbal tea	*In vivo* experiment: mouse modeling, group experiment, drug treatment		*Bacteroides↑* *Lactobacillus↑* *Bifidobacterium↑*	(75)	
Polyphenols (catechins) in oolong tea	*In vitro* experiments: Stimulation studies were conducted using *in vitro* batch culture models of the distal region of the human large intestine		*Enterococcus↑* *Bifidobacterium↑* *Lactobacillus↑*	(68)	
Red wine polyphenols (procyanidins)	*In vitro* fermentation experiment		*colibacillus↑ Blautia coccoides↓* *Klebsiella↑ Bacteroides↓* *Alistipes↑* *Bifidobacterium Subdoligranulum↓* *Akkermansia↑ C. coccoides↓* *Victivallis ↑ Bifidobacterium↓*	(68)
Polyphenols (gallic acid and caffeic acid) in fermented papaya juice	The patients were supplemented with tube feeding, and the pH value of the colon was analyzed		*Against↓* *Bacteroides↓* *Clostridium perfringens↓* *C. difficile↓* *Bifidobacterium Eubacteria↑*	(76)
Polyphenols(procyanidins) in grape seeds	*In vivo* experimental addition to the pig diet		*Lactobacillus, Clostridium↑, and Ruminococcus↑*	(61)
Blueberry polyphenols (chlorogenic acid and malvidin 3, 5-diglucoside)	*In vitro* study using Campylobacter Organisms (CLO test) and blood agar plating		*H. Pylori↓*	(77)
Carrot dietary polyphenols	*In vivo* experiments: *In vitro* incubation using male BALB/c mice and *in vitro* human fecal samples		*Bacteroides*, *Lactobacillus and lower Proteobacteria (Clostridiales, Ruminococcus, Coprococcus, Oscillospira)*	(62)
Lentil polyphenol (cysteamine)	AGAR plate diffusion method was used		*coli↓* *Staphylococcus aureus↓*	(78)
Polyphenols (flavan-3-ols and chlorogenic acid) in coffee	*In vivo*: male Wistar rats were fed coffee grounds		*Colonies of Ruminococcaceae↑ Muribaculaecae Trichomillillaceae↑* *Firmicutes and Bacteroidetes↓*	(79)
Nut polyphenols (ellagitannins and proanthocyanidins)	Analysis of plasma circulation in consumers		*Bifidobacterium Lactobacillus↑*	(80)
Resveratrol	*In vivo* experiments: Wistar rats were divided into groups and fed an HFS diet supplemented with resveratrol (15 mg/kg body weight (BW)/day).		*Firmicutes/Bacteroidetes ratio↓ Erysipelotrichaceae Bacillus↓* *Eubacterium cylindroides↓*	(81)

"↑" : The number is increased; "↓" : The number is reduced.

## 3 Synergistic effect of polyphenols and GM to treat OP

### 3.1 The role of polyphenols in the treatment of osteoporosis

In addition to short-chain fatty acids, polyphenols can be metabolized into different substances, such as phenolic acid, glucuronic acid, sulfate, etc.

Apigenin, a flavone commonly found in fruits and vegetables, can be metabolized into p-coumaric acid by gut microbiota. The metabolism of apigenin by the gut microbiota highlights the role of these microorganisms in breaking down dietary compounds and generating metabolites with potential health benefits. p-coumaric acid itself possesses antioxidant and anti-inflammatory properties, and its production through apigenin metabolism adds to the overall beneficial effects of flavonoid consumption on human health (82). In cell line studies, 4-hydroxycinnamic acid exhibited notable anti-inflammatory activity in LPS-stimulated macrophage cells. Specifically, it was observed to inhibit the activity of nitric oxide synthase (iNOS), an enzyme involved in the production of nitric oxide, which plays a role in inflammation. This suggests that 4-hydroxycinnamic acid may have potential as an anti-inflammatory agent by modulating the iNOS pathway in immune cells. Nevertheless, it is essential to acknowledge that additional research is required to corroborate these findings and investigate the possible therapeutic uses of 4-hydroxycinnamic acid.

Research conducted on rat femoral tissue showed that 4-hydroxycinnamic acid increased calcium content and affected bone metabolism *in vitro*. These findings suggest that 4-hydroxycinnamic acid holds potential benefits for osteoporosis and overall bone health. However, it is important to acknowledge that findings from *in vitro* studies offer preliminary evidence, and further research, including *in vivo* studies and clinical trials, is necessary to confirm these effects in humans. Further research is required to validate these findings in animal models and human clinical trials to fully understand the effects of 4-hydroxycinnamic acid on bone health (83).

Research has shown that daidzein, a key soy isoflavone in our diet, can be converted to equol by certain gut microorganisms. This conversion has been linked to positive health effects in individuals who produce equol. In women with osteopenia, taking red clover extract (RCE) with probiotics twice daily for a year has been found to effectively reduce bone mineral density loss caused by estrogen deficiency. Additionally, phlorizin, a natural compound found in several fruit trees, is a dietary component (84). Its metabolites are Phloretin (phloretic acid and phloroglucinol). Phloretin and its derivatives, primarily in glycosyl forms, are naturally occurring dihydrochalcones found in fruits like apples, kumquat, pear, strawberry, and various vegetables (85).

The osteoprotective effects of phloretin, a dihydrochalcone present in apple tree leaves, were examined in ovariectomized (OVX) C57BL/6 female mice to assess its potential for preventing bone loss (86). The researchers discovered that phloretin modulated the ASK-1-MAPK signal transduction pathway, resulting in the transcription of apoptotic genes. This mechanism effectively prevented osteoclast absorption induced by estrogen deficiency, thereby highlighting the potential of phloretin in mitigating bone loss (87). In conclusion, phloridzin metabolites play an important role in regulating bone dynamics and increasing bone mineral density and content.

Genistein is indeed a secondary metabolite commonly found in leguminous plants, seeds, fruits, and vegetables. It belongs to the class of compounds known as isoflavones and exhibits phytoestrogenic activity. Genistein, a phytoestrogen, can mimic the structure or function of 17β-estradiol, a naturally occurring estrogen in mammals. Several studies have indicated that higher dietary intake of phytoestrogens like genistein is associated with increased bone mineral density (BMD) in postmenopausal women, as observed in cross-sectional analyses. However, it’s worth noting that these effects were primarily observed in postmenopausal Chinese women and not in premenopausal women. The exact mechanisms through which genistein influences bone health are still being investigated. It is believed that genistein may modulate the estrogen receptor pathway and exert estrogen-like effects on bone tissue, leading to potential benefits for bone density. Indeed, additional research, including prospective studies and clinical trials, is necessary to gain a more comprehensive understanding of the association between genistein consumption and its effects on bone health in diverse populations (88).

In studies conducted on ovariectomized (OVX) rats, genistein administered orally at a dose of 10 mg/kg for 12 weeks has been shown to stimulate bone formation and possess inhibitory properties against bone resorption (89).

Most of the intestinal metabolites of polyphenols have anti-inflammatory and antioxidant effects, so they have an important role in the treatment of osteoporosis.

#### 3.1.1 Anti-osteoporosis mechanism of polyphenols

##### 3.1.1.1 Anti-oxidative stress

Oxidative stress occurs when there’s an imbalance between the production and elimination of reactive oxygen species (90, 91). Excessive reactive oxygen species can cause cell damage and apoptosis, affect cell function, and trigger diseases (74). Oxidative stress can affect the functioning of bone marrow-derived mesenchymal stem cells, thereby influencing both bone growth and osteogenic differentiation of mesenchymal stem cells. Consequently, this can result in impaired osteoblast function and accelerated formation and differentiation of osteoblasts (92) (93).

However, the presence of antioxidants can provide cellular protection against damage induced by reactive oxygen species. Polyphenolic compounds contain a large number of phenolic hydroxyl groups that act as hydrogen donors to reduce singlet oxygen to less active triplet oxygen, thereby reducing the probability of oxygen radical generation and terminating chain reactions triggered by free radicals (94). In addition, they can scavenge free radicals and protect biological macromolecules from free radical damage (95). There is research evidence that the intake of natural berries rich in foodborne polyphenolic compounds, such as cranberries and blueberries, can combat oxidative stress by scavenging free radicals, and prevent and treat osteoporosis (96).

##### 3.1.1.2 Anti-inflammatory effects

Polyphenolic compounds exert anti-inflammatory effects by negatively regulating inflammatory pathways, especially their regulation of the key NF-κB transcription factor (TF) (97, 98). Estrogen receptors are capable of engaging in protein interactions with NF-κB, leading to the formation of complexes and subsequent binding of NF-κB to specific response elements. These specific response elements regulate the transcription of NF-κB-dependent genes in a cell type-specific manner and are crucial in modulating inflammatory processes (99).

For example, TP can inhibit lipid peroxidation and combat oxidative stress by regulating the transcription factor NF-κB and acting as an estrogen receptor ERK in HMC-1 cells. Impaired expression of inducible nitric oxide synthase reduces the production and release of inflammatory factors TNF-1, IL-6, IL-8, and NO. This process, in turn, brings about anti-inflammatory effects and helps mitigate bone loss (100).

In addition, prong and its polyphenolic compounds have been shown to inhibit bone resorption by down-regulating the receptor activated NF-κB ligand (RANKL), and to directly inhibit the generation of osteoblasts by down-regulating NFATc1 and inflammatory mediators, thereby reducing osteogenic activity (101, 102). Under lipopolysaccharide (LPS) induced inflammatory conditions, the expression of cyclooxygenase and the production of nitric oxide (NO) in osteoblastic progenitors were inhibited by polyphenols extracted from plums at concentrations of 10, 20, and 30μg/mL. The inhibition was achieved by downregulating the expression of inducible nitric oxide synthase (103). In the presence of RANKL, these polyphenolic compounds simultaneously stimulated bone formation and suppressed the generation of NO and tumor necrosis factor (TNF)-α (104, 105). TNF-α production increased over time in response to oxidative stress stimulation, and dried plum polyphenols were able to reduce the differentiation of bone resorptive cells under normal conditions as well as under inflammatory and oxidative stress conditions (106).

##### 3.1.1.3 Activate the Wnt/β-Catenin pathway

The Wnt signaling pathway plays a critical role in both bone development and the maintenance of metabolic homeostasis (107). Wnt-related proteins or factors can bind to the Frizzled gene receptor (Fzd) and initiate downstream intracellular cascade reactions, thereby regulating the transcription or expression of target genes such as β-Catenin, peroxisome proliferator-activated receptor γ (PPARγ), and RUNX2 (Runt-related transcription factor 2) (108, 109). Then it regulates the physiological processes of osteoblast formation, differentiation, and maturation.β-Catenin is a pivotal factor in the classical pathway, serving as a central regulator of the Wnt/β-Catenin signaling pathway. β-Catenin can enhance the activity of alkaline phosphatase (ALP) while promoting bone. Runx2 is a specific transcription factor of osteoblasts, which is closely related to the proliferation and differentiation of osteoblasts.

For example, icariin and resveratrol have been widely used in the prevention and treatment of OP (110). Potential applications for regulating the osteogenic differentiation of BMSCs, preventing bone loss, and promoting bone regeneration have been discovered. In a study by Wei et al., it was found that icariin intervention in rat bone marrow stromal cells increased total β-catenin and nuclear translocation by stimulating β-catenin activation. Additionally, the expression of Wnt signaling members (β-catenin, Lef1, TCF7, c-jun, c-myc, and cyclin D) was significantly upregulated (111). Moreover, the activation of ERα was found to enhance the expression of osteogenic genes, thereby promoting both the proliferation and osteogenic differentiation of BMSCs. Similarly, resveratrol is one of the effective active components of Polygonum knotweed and veratrol and has estrogen-like effects. Some researchers concluded by intervening in OVX rats through the Wnt/β-catenin pathway mediated by resveratrol (112). By stimulating the expression of Runx2, resveratrol down-regulates the expression level of GSK38, preventing the effective formation of β-catenin degradation complex, ensuring the stable accumulation of β-catenin in cytoplasm and translocation to the nucleus, thus activating Wnt/B-catenin pathway, promoting osteogenesis differentiation and enhancing bone density, playing a role in preventing and treating OP.

##### 3.1.1.4 Inhibition of the NF-κB pathway

The NF-κB signaling pathway has a significant role in bone metabolism and can also interact with other signaling pathways to impact the progression of osteoporosis (113, 114). The NF-κB signaling pathway is mainly composed of IκB protein, core IκB kinase (IKK) complex, and NF-κB. The IκBα proteasome degrades the translocation signal of exposed NF-κB/p65 subunits, promoting NF-κB to enter the nucleus and bind to related genes, initiating transcription of these genes. The NF-κB pathway regulates bone metabolism and influences the skeletal system.

**Figure 3:**
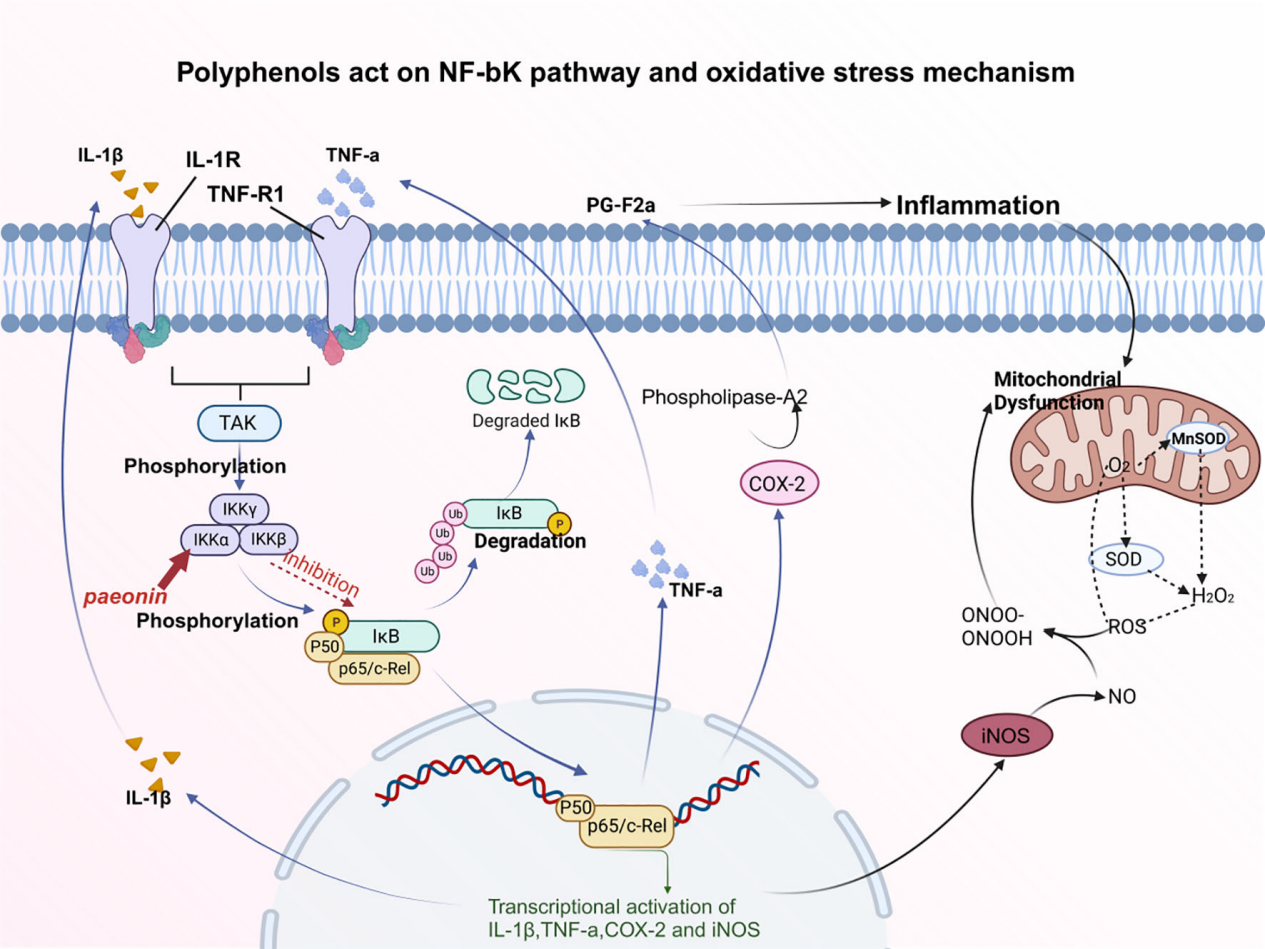
Polyphenols act on the NF-κB pathway and the mechanism of oxidative stress. Paeoniflorin weakens the phosphorylation level of p65 NF-κB, thereby inhibiting the activation of NF-κB signaling pathway. Oxidative stress is closely related to inflammatory cytokines, which are always associated with the NF-κB pathway. Inflammation can cause mitochondrial dysfunction, which prevents oxidative metabolism. After the inhibition of the NF-κB signaling pathway, PG-F2α can be down-regulated to inhibit inflammatory factors.

For example, Lin et al. applied paeoniflorin to intervene osteoclast model differentiated from RAW 264.7 cell line to observe the effect of paeoniflorin on the osteoclast signaling pathway. The results suggested that paeoniflorin weakened the phosphorylation level of p65 NF-κB, that is, inhibited the activation of the NF-κB signaling pathway (115, 116). The NF-κB pathway is one of the main biological pathways of osteoclast differentiation (117). It has been verified that paeoniflorin reduces the activity of the NF-κB signaling pathway, reduces the activity of osteoclasts, reduces bone resorption, and further maintains bone homeostasis by inhibiting the activation of p65 NF-κB (118). What’s more, Wang used ostiole to interfere with osteoclasts and studied the mechanism of action of OST on osteoclasts (119). The experimental findings demonstrated an up-regulation in the expression of P65 NF-κB, while down-regulation was observed in the expression of NFATc1, CTSK, MMP-9, TRAP, and p-IκB., the expression of the NF-κB pathway can inhibit the further differentiation of osteoclasts, and the activity gap between osteoblast and osteoblast can be narrowed to a large extent. This is the molecular mechanism of antiosteoporosis of OST via the NF-κB pathway. Both peony and snake seeds are traditional Chinese medicine and contain polyphenols. By inhibiting the NF-κB signaling pathway, the production of osteoclasts is inhibited, to achieve the therapeutic effect of osteoporosis (116) **Figure 3**.

#### 3.1.2 Polyphenols promote bone formation and inhibit bone absorption

Researchers have extensively studied the beneficial effects of polyphenols, which are known to enhance bone formation and suppress bone resorption. During the process of bone formation, osteoblasts play a vital role in the synthesis and secretion of crucial components of the bone matrix, including collagen and glycoproteins (120). Through the study of MC3T3-E1 cells, SaOS-2 cells, D1 cells, NRG cells, osteosarcoma cells, and other cell models, it has been confirmed that tea polyphenols can enhance the activity of alkaline phosphatase (ALP) (121), increase bone mineral formation and bone mineralization area, improve bone mineral density, and thereby promote bone formation. Bone resorption occurs when hematopoietic stem cell-derived pre-osteoblasts transform into osteoblasts due to the presence of M-CSF, RANKL, and cytokines (122). These cytokines have the ability to induce osteoblasts to undergo cell polarization, leading to their active involvement in the process of bone resorption.

According to a study, it was found that MGF has the ability to impede the differentiation process of pre-osteoblastic macrophages (BMM) induced by M-CSF and RANKL, preventing their transformation into TRAP-positive multinucleated macrophages (123), i.e. osteoblasts, suggesting that MGF could inhibit the differentiation of BMM macrophages and promote the expression of ER-β mRNA. MGF promotes the proliferation and differentiation of osteoblast precursor cells MC3T3-E1 through RunX2; therefore, MGF may promote bone formation of osteoblasts, thereby regulating the balance of osteoblast and bone resorption cell functions (124).

### 3.2 The role of GM in the treatment of OP

Recent research has revealed a significant link between gut microbiota and osteoporosis. Gut microbiota regulates bone homeostasis and can affect osteoporosis through various mechanisms. These include the modulation of its metabolites, influencing host metabolism, altering drug metabolism, and regulating the integrity of the gut barrier function (125). Numerous studies have highlighted alterations in the collagen properties of the gut microbiota, which are closely linked to bone fragility. These changes encompass variations in biochemical properties and protein structure, emphasizing the significant role of the gut microbiota in bone health. Supplementation with specific probiotics in mouse models associated with osteoporosis improved bone density and enhanced bone heterogeneity. In addition, quercetin fights osteoporosis by regulating the level of short-chain fatty acids (SCFAs), improving the bone microenvironment, and restoring the integrity of the intestinal mucosa (126). Another study by Zhang et al. showed that fecal flora transplantation (FMT) improved bone loss in osteoporosis mice after ovariectomy by regulating gut microbiota and metabolic function (127).

The gut microbiome plays a crucial role in maintaining bone health by influencing the immune system, which is closely connected to bone cells. It achieves this by utilizing the host’s fully developed immune system to regulate responses throughout the body, thus controlling bone turnover and density. The gut microbiota improves bone health, enhances calcium absorption, and regulates serotonin production in the gut, which interacts with bone cells and is considered a bone regulator (128).

The gut microbiota initially varies but stabilizes quickly as the immune system responds to environmental factors. The composition of the gut microbiota changes with age, with great variability in the elderly (>65 years) (129). The gut microbiota offers many possible antigens for the immune system of the host. Under normal conditions, a harmonious relationship exists between the host and the commensal bacteria, which aid in food digestion and protect against intruding pathogens (130). In certain conditions where the host’s ability to control the entry of gut microbiota is compromised, certain species may invade host tissues and cause disease. Changes in the composition of gut microbiota can lead to intestinal inflammation and disrupt the balance of the immune regulatory network, which has been linked to osteoporosis in numerous studies (131).

In addition, gut microbiota alleviates oxidative stress by producing antioxidant molecules such as glutathione, folate, and polysaccharides (132, 133). Furthermore, certain components of the intestinal microbiota have the capability to produce short-chain fatty acids (SCFA). These SCFAs not only stimulate the generation of antioxidant molecules but also aid in mitigating oxidative stress (134).

Certain lactic acid bacteria in the gut have been found to aid in preventing osteoporosis by reducing mutagenic activity. These bacteria can attach themselves to potent mutagens in the gut, lessening their mutagenic impact. This, in turn, lowers the levels of inflammation and DNA damage, thus providing superior shielding for the gut wall. Furthermore, this process promotes improved mineral absorption, ultimately thwarting the onset of osteoporosis (135).

In addition, exopolysaccharides exhibit a vast variety of biological activities, such as immunomodulatory, antioxidant, anti-tumor, and regulation of intestinal microbial balance, thereby improving immune response and playing an anti-inflammatory and antioxidant role (136).

The study examined how Lactobacillus plantarum extracellular polysaccharide affects the intestinal immune response, oxidative stress, intestinal mucosal barrier, and microbial community in immunosuppressed mice induced by cyclophosphamide (137). These results suggest that the extracellular polysaccharide of L. plantarum JLAU103 may regulate the intestinal immune response by regulating SCFA production and intestinal microbiota in immunosuppressed mice, thereby activating systemic immunity (137). In a separate study, Bifidobacterium WBIN03 was identified as having a high growth rate and exopolysaccharide production. The effects of these exopolysaccharides on the intestinal microflora in mice were examined. The study showed that exopolysaccharides boosted the growth of Lactobacillus and anaerobic bacteria while suppressing Enterobacter, Enterococcus, and Bacteroides fragilis (138). An additional analysis of the gut microbiome revealed that Lactobacillus plantarum NCU116 enhanced the abundance of microbial populations involved in gut regeneration and glycan metabolism (139).

In conclusion, in addition to their metabolites, their exopolysaccharides also have anti-inflammatory and antioxidant stress effects and also have a certain impact on the treatment of osteoporosis.

### 3.3 Effects of GM improved by PPs on OP treatment

Due to their capacity to inhibit inflammatory factors and engage in various other mechanisms, polyphenols have been identified as potential agents for the treatment of osteoporosis, and chronic inflammation, and multiple mechanisms are closely intertwined with the function of the intestinal barrier, the gut microbiota is usually related to the immune regulatory network, and the regulation of the immune system is often induced by inflammatory factors, and inflammation is closely related to bone loss and osteoclast activation (140). Osteoporosis is treated by converting polyphenols into metabolites to inhibit inflammatory factors, enhance intestinal barrier function, and regulate immunity to inhibit bone loss and osteoclast formation (141). Meanwhile, polyphenols can increase the abundance and activity of the gut microbiota, acting as regulatory mediators and inducers in the gut barrier-bone-immune system (142). Studies in mice with osteopenic ovariectomies fed a diet supplemented with crude extracts of dried plums and dried plums polyphenol compounds showed that the polyphenols caused modifications in both the gut microbiota and the levels of cecal short-chain fatty acids. These findings demonstrate the potential prebiotic activity of dried plum polyphenols and their significant contribution towards regulating both bone formation and bone resorption (143). Sangeeta Huidrom et al. conducted a study, which demonstrated that the oral administration of various strains of probiotics exhibited promising effects in reducing bone resorption and increasing bone density. This finding was observed in both animal models and human studies, suggesting the potential of probiotics as a therapeutic approach for osteoporosis (144). Therefore, probiotics may be an effective way to prevent and treat postmenopausal osteoporosis. As a prebiotic, polyphenols can improve gut microbiota and increase the number of intestinal probiotics, to achieve the effect of treating osteoporosis (144). Hence, the synergistic effect between polyphenols and gut microbiota emerges as a critical factor in the treatment of osteoporosis.

## 4 Conclusions and perspectives

Osteoporosis is a common metabolic disease. In this paper, the effects of polyphenols and intestinal microbes on the treatment of osteoporosis were summarized. Polyphenols can be decomposed into metabolites that are more easily absorbed, and the abundance and activity of intestinal microorganisms are increased due to the action of polyphenols. Under the synergistic effect of the two, they play their respective functions and roles to a greater extent, providing innovative ideas and important insights for the treatment of osteoporosis.

## Author contributions

KW: Writing – original draft, Writing – review & editing. HS: Writing – original draft, Writing – review & editing.
